# Deep Reinforcement Learning-Based Task Scheduling in IoT Edge Computing

**DOI:** 10.3390/s21051666

**Published:** 2021-02-28

**Authors:** Shuran Sheng, Peng Chen, Zhimin Chen, Lenan Wu, Yuxuan Yao

**Affiliations:** 1 School of Information Science and Engineering, Southeast University, Nanjing 210096, China; shengshuran@seu.edu.cn (S.S.); wuln@seu.edu.cn (L.W.); 2 State Key Laboratory of Millimeter Waves, Southeast University, Nanjing 210096, China; 3 School of Electronic and Information, Shanghai Dianji University, Shanghai 201306, China; chenzm@sdju.edu.cn; 4 Shannxi Key Laboratory of Integrated and Intelligent Navigation, Xi’an 710068, China; yyx_13022958068@163.com

**Keywords:** Internet of Things (IoT), edge computing, task scheduling, markov decision process (MDP), deep reinforcement learning (DRL)

## Abstract

Edge computing (EC) has recently emerged as a promising paradigm that supports resource-hungry Internet of Things (IoT) applications with low latency services at the network edge. However, the limited capacity of computing resources at the edge server poses great challenges for scheduling application tasks. In this paper, a task scheduling problem is studied in the EC scenario, and multiple tasks are scheduled to virtual machines (VMs) configured at the edge server by maximizing the long-term task satisfaction degree (LTSD). The problem is formulated as a Markov decision process (MDP) for which the state, action, state transition, and reward are designed. We leverage deep reinforcement learning (DRL) to solve both time scheduling (i.e., the task execution order) and resource allocation (i.e., which VM the task is assigned to), considering the diversity of the tasks and the heterogeneity of available resources. A policy-based REINFORCE algorithm is proposed for the task scheduling problem, and a fully-connected neural network (FCN) is utilized to extract the features. Simulation results show that the proposed DRL-based task scheduling algorithm outperforms the existing methods in the literature in terms of the average task satisfaction degree and success ratio.

## 1. Introduction

Technology advancements in sensing, communications, and computing directly accelerate the recent development of the Internet of Things (IoT), leading to diverse IoT uses [1,2]. Most IoT-enabled applications are computationally-intensive, such as interactive gaming and augmented reality (AR) [3], and it is difficult for the devices themselves to fulfill such tasks due to the hardware constraints and power consideration. One feasible solution is to offload the tasks to the remote cloud for processing and return the results to the end devices. Although the cloud servers provide sufficient computation resources, a large amount of traffic delivered to the cloud would result in network congestion and unpredictable delay, which fails to meet the low latency requirement and degrades the quality of experience (QoE). The emerging edge computing technology overcomes the shortcomings of cloud computing [4,5].

Mobile edge computing enables various IoT applications and services performed at the network edge instead of being delivered to the remote cloud, which reduces the response time and alleviates the burden on the backhaul link. With edge computing, computationally-intensive IoT tasks are sent to the nearby VMs configured at the edge server to achieve low latency services [6,7,8]. However, the computation, storage, and network resources of the edge server are limited, and thus, task scheduling is vital to maximize the quality of experience (QoE) [9,10]. Task scheduling in edge computing is more challenging due to several aspects. First, the transmission delay is stochastic due to the dynamic wireless channel condition or network environment between the end devices and the edge node. Second, the available resources provided by the VMs are different in terms of their speed, ready time, and response time. Lastly, the task arrival rate, task size, and delay requirement are diverse for various IoT applications, making task scheduling in edge computing more challenging.

Two special problems must be addressed for task scheduling in edge computing: time scheduling and resource allocation. Time scheduling determines the task execution order, and resource allocation is responsible for assigning tasks to suitable virtual machines (VMs) for execution. A number of task scheduling aspects in edge computing have been studied [11,12,13,14,15,16]. However, most existing works aim at resource allocation, while time scheduling has been seldomly studied. In [17], a general online scheduling model was proposed to minimize the task response time when tasks are offloaded to the edge servers. Based on Lyapunov optimization, a scheduling algorithm was proposed in [18] to minimize the communication delay and computing delay. In [19], a dual-scheduling framework in heterogeneous vehicular edge computing was proposed to adapt to the unstable capacity of servers and the task arrival rate. In [20], computationally-intensive data-parallel task offloading and scheduling were realized based on the first-come-first-serve (FCFS) mechanism to minimize the average completion time through a mixed integer non-linear programming (MINLP) algorithm. In [21], the shortest-job-first (SJF) scheduling method was applied in the task scheduling, where the task with the minimum delay is scheduled first. The authors in [22] investigated device-to-device (D2D) collaboration for task offloading by taking into account human mobility to optimize the task assignment and power allocation. In [23], the joint optimization problem of task allocation and the time scheduling problem were formulated as mixed-integer programming (MIP), and the logic-based Benders decomposition (LBBD) approach was proposed to maximize the number of admitted tasks. A heuristic algorithm was proposed in [24] to address the energy-efficient and delay-sensitive task scheduling in IoT edge computing. In [25], the task scheduling and dispatching of networking and computing resources were investigated to maximize the number of completed tasks. These methods are based on an ideal mathematical model and optimized by a mixed-integer non-linear programming (MINLP) or heuristic algorithms. Although these model-oriented algorithms can achieve good results, they are not adapted to the dynamic environment where the task arriving rate and popularity are unknown in advance. Furthermore, the model-based task scheduling algorithms focus on the one-step optimization rather than on the long-term objective. These algorithms assume the availability of resources is fixed during the scheduling period.

The Markov decision process (MDP) is an effective approach to model the sequential decision-making problem to achieve a long-term objective. Reinforcement learning (RL) has been developed as a promising approach to solve the MDP problems, where the agent makes sequential decisions by continually interacting with the environment [26,27]. The ultimate goal of the agent is to find an optimal policy to maximize the cumulative reward instead of the local optimal solution in real time. In RL, the mapping between the state and action is stored in a tabular form, which is not practical, especially for the large state space and continuous action space. Combined with the deep neural network (DNN), model-free deep reinforcement learning (DRL) is capable of making intelligent sequential decisions in sophisticated environments, and the table in RL is hence replaced by the function approximation of the DNN.

In recent years, DRL has been successfully applied to time scheduling and resource allocation in edge computing [28,29,30]. The computation resource allocation problem in edge computing is formulated as an MDP, and multiple replay memories were utilized for the deep Q-network (DQN) algorithm to minimize the total delay and resource utilization [31]. In [32], a DQN-based task scheduling was studied in cloud computing to maximize the number of successful tasks by considering the delay requirement. The authors in [33] investigated joint task offloading and resource allocation for computationally-intensive tasks in fog computing. The problem was formulated as a partially observable MDP, and the deep recurrent Q-network (DRQN) algorithm was applied to approximate the optimal value functions. In [34], a reinforcement learning algorithm was explored to address the delay-optimal task scheduling problem in cloud computing. In [35], a DRL-based approach was proposed to address the task scheduling and offloading problems in vehicular edge computing, while the latency demands were not considered. In [36], task scheduling with multiple resource allocation problems was tackled with DRL and imitation learning, where two objectives were defined.

In this paper, we design an intelligent task scheduling framework in edge computing. We focus on the heterogeneous VM resources for the task scheduling to maximize the long-term value of the QoE by considering the expected delay requirement. In achieving this goal, the DRL algorithm is applied, and the task satisfaction degree is determined as the reward. The action of the mechanism consists of two parts: one is determining the task execution order, and the other is assigning the task to the suitable VM. We formulate the task scheduling process in edge computing into an MDP, which is solved by a policy-based DRL algorithm. The main contribution of this article can be summarized as follows.

- Model-free DRL-based task scheduling is studied for task scheduling in edge computing, where the time scheduling and VM assignment are jointly optimized. The problem is formulated as an MDP problem, where the availability of VMs, task characteristics, and queue dynamics are taken into account.
- The action is represented as a VM-task pair, whose dimension may be extremely large. A new mechanism is designed in the MDP formulation, where the scheduling time step is decoupled from the real time step. By this mechanism, the action space stays linear with the product of the number of VMs and the queue size, and multiple tasks can be scheduled in one time step.
- Extensive simulation results demonstrate that the proposed DRL-based algorithm achieves a better task satisfaction degree in comparison with the baseline task scheduling algorithms.

The remainder of the paper is organized as follows. The system is presented in Section 2. In Section 3, the task scheduling in the edge computing problem is formulated as an MDP, and then, the DRL-based algorithm is applied. The simulation of the evaluation results is given in Section 4. Finally, the conclusions are given in Section 5.

## 2. System Model

In this section, the system architecture of the task scheduling in edge computing is introduced first, then the task model, task scheduling mechanism, and overall optimal objective are elaborated. Some notations are listed in Table 1.

### 2.1. System Architecture

**Figure 1:**
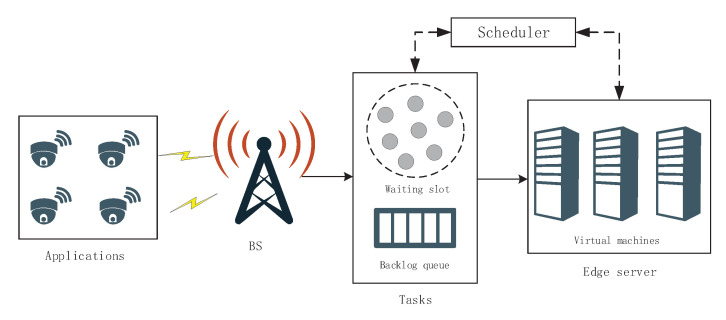
Illustration of the system model.

We consider a task scheduling framework in an edge computing system, as illustrated in Figure 1. The computationally-intensive tasks generated by IoT applications, which are difficult to perform at local devices, are delivered to the server, which is deployed at the network edge close to the end devices. The edge server is configured with several VMs, which vary significantly in their computational capacity and ready time to execute the next scheduled task. After arriving at the edge server, the tasks wait to be scheduled.

For simplicity, we only focus on the computational resource for task scheduling. The scheduler monitors the status information of incoming tasks and the VMs that have an impact on the scheduling decision-making, including the task sizes, the expected completion time, the computing speed (in million instructions per second (MIPS)), and the waiting time. Based on the observation, the scheduler makes decisions on when to schedule (i.e., the scheduling order and the start time of each task) and where to schedule (i.e., which VM is allocated to each task). The tasks waiting to be scheduled are divided into two sets: one is the waiting set inside the circle in Figure 1, and the other is stored in the backlog queue. Each task of the waiting set occupies a waiting slot that can be fully observed, while only the number of tasks in the backlog queue can be observed by the scheduler. At each scheduling time step, the scheduler selects at most one task in the waiting slot to schedule. In this article, we investigate task scheduling in edge computing for which only one edge server is deployed. The objective is to maximize the long-term task satisfaction of all tasks, which is:

(1) $$max\sum_{t=1}^{T}\sum_{i\in J,j\in V}g_{i,j},$$

where $g_{i,j}$ is the task satisfaction of the task *i* scheduled to VM *j*. To achieve the objective, we need to model from the following aspects.

### 2.2. Task Model

Computationally-intensive tasks arrive at the edge server dynamically and are classified into K types, $J=\{j_{1},j_{2},...,j_{K}\}$. It is assumed that the tasks belonging to the same type have the same characteristics, including the task size (million instructions (MI)) and the delay requirement. The task types are ranked in ascending order by task size, and the popularity of the tasks is characterized by the Zipf distribution with the parameter popularity skewness β as $p_{j}=j^{-\beta }/\sum_{j=1}^{C}j^{-\beta }$. Therefore, a task *i* belonging to one of the *K* types can be denoted by a tuple as:

(2) $$j_{i,k}=\langle a_{i},z_{i},l_{i},d_{i}\rangle ,$$

where $a_{i}$, $l_{i}$, and $d_{i}$ are the arriving time, the size, and the expected delay of the task $j_{i}$, respectively.

### 2.3. Task Scheduling Mechanism

The edge server is configured with several VMs, denoted by $V=\{v_{1},v_{2},...v_{M}\}$. These VMs are heterogeneous in terms of their computing capacity, denoted by $C=c_{1},c_{2},...,c_{M}$. The task scheduler decides how to schedule tasks: determine the scheduling order and to which VM to assign. When a task is scheduled, it leaves the waiting slot, and the first task stored in the backlog queue is put into the waiting slot just vacated. It is assumed that each task is only processed on a single VM and that the computation resource of the VM will be fully utilized. The expected processing time in each VM is known before its execution. When a task is scheduled to a VM, its response time includes the waiting time in the waiting slot and the VM execution time. The execution time of task *i* in VM *j* can be computed as:

(3) $$e_{i,j}=\frac{l_{i}}{v_{j}}.$$

If no tasks are executed by the VM, the start time of the current task is the arriving time; otherwise, the task begins being executed when the VM is available, i.e., all the earlier tasks have been finished. Let $s_{i,j}$ and $f_{i,j}$ denote the starting time and finishing time of task *i* on VM *j*. Therefore, the starting time depends on the finishing time of all the last tasks, which can be expressed as $s_{i,j}=max\left\{f_{l,j},a_{i}\right\}$, and the task finishing time of task *i* can be calculated as:

(4) $$f_{i,j}=s_{i,j}+e_{i,j},$$

where $e_{i,j}$ is the time for VM *j* to process task *i*. The response time of task *i* on VM *j* is composed of two parts: the waiting time and the execution time:

(5) $$t_{i,j}=w_{i,j}+e_{i,j},$$

where $w_{i,j}$ is the waiting time of task *i* on VM *j*. If the task is processed immediately, there is no waiting time; otherwise, it is the time gap between the starting time and the arriving time and is given as:

(6) $$w_{i,j}=s_{i,j}-a_{i}.$$

The response time is applied to evaluate the QoE of the tasks. For each task, the QoE is defined as the task satisfaction degree, which is the ratio of the expected latency and the response time. The task satisfaction degree of the task executed on VM *j* can be represented as:

(7) $$g_{i,j}=d_{i}/t_{i,j},$$

where $d_{i}$ is the expected latency. It is obvious that the larger the ratio, the higher the task satisfaction degree is.

## 3. DRL Solution

The task scheduling problem is addressed by the model-free DRL according to the Markov decision process (MDP), which is an efficient mathematical model to model the sequent decision-making problem in a dynamic environment. This section gives the carefully designed MDP, and the policy-based DRL algorithm is applied to solve the task scheduling problem.

### 3.1. Preliminaries

DRL is an effective approach to deal with the Markov decision process (MDP) with a large-scale state space and action space. The ultimate goal of the DRL algorithm is to find an optimal policy $\pi^{*}$ to maximize the expected return (long-term cumulative reward), which is considered as the state value function *V*. A sequence of decisions is made through the continuous interaction of the agent with the unknown environment. At the time of the scheduling time step *n*, the value function *V* under a policy π can be represented as [37]:

(8) $$V_{\pi }=E_{\pi }\left[G^{n}\right]=E_{\pi }\left[r^{n}+\gamma r^{n+1}+\gamma^{2}r^{n+1}+\cdots \right],$$

where γ∈[0,1] is a discounted factor, showing how important the future rewards are to the cumulative return, *r* is the instant reward obtained at each timestep, and $E\left[\cdot \right]$ is the expectation operator.

**Figure 2:**
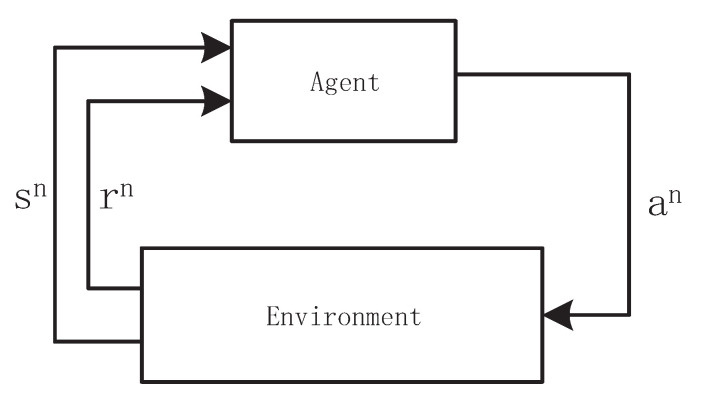
Interaction between the agent and the environment.

In each interaction, the agent takes action based on the observed state $s^{n}$, then it receives a feedback reward *r* and a new state from the environment, as shown in Figure 2. The action-state value function of a state-action pair, namely the Q-function, is defined as:

(9) $$\begin{array}{cc} Q_{\pi }\left(s,a;\theta \right) & =E_{\pi }\left[G^{t}|s^{n}=s,a^{n}=a\right]\\ \; & =E_{\pi }\left[\sum_{k=n}^{\infty }\gamma^{k-n}r^{k}|s^{n}=s,a^{n}=a\right], \end{array}$$

in which θ is the DNN paramter. Then, we have the optimal value:

(10) $$V_{*}\left(s\right)=\max_{\pi }V_{\pi }\left(s\right),Q_{*}\left(s,a\right)=\max_{\pi }Q_{\pi }\left(s,a\right),$$

and the optimal policy:

(11) $$\pi_{*}=\underset{\pi }{\arg\max}V_{\pi }\left(s\right),\pi_{*}=\underset{\pi }{\arg\max}Q_{\pi }\left(s,a\right).$$

The goal of the DRL is to find an optimal behavior strategy for the agent to obtain optimal rewards. The optimal policy can be achieved by two methods: the value-based method and the policy-based method. The value-based methods aim to learn the Q function and then select an action with the maximum value, $\hat{a}=\arg\max_{a\in A}Q\left(s,a\right)$. The policy gradient methods instead target modeling and optimizing the policy $\pi_{\theta }\left(a|s\right)$ directly with a parameterized function with respect to θ [38]. In the policy gradient, the action is chosen following $\pi_{\theta }\left(a|s\right)$, which is a distribution of action probabilities with the softmax function:

(12) $$\pi_{\theta }\left(s,a\right)=\frac{e^{\phi {\left(s,a\right)}^{T}}\theta }{\sum_{k=1}^{K}e^{\phi {\left(s,a_{k}\right)}^{T}}\theta },$$

where ϕ(s,a) is the feature vector.

Compared with value-based methods, policy gradient methods directly predict the action and naturally explore it due to its stochastic policy representation. Moreover, it is more effective in high-dimensional or continuous action spaces. The objective of the policy gradient algorithm is:

(13) $$\begin{array}{cc} J(\theta ) & =\sum_{s\in S}d_{\pi_{\theta }}\left(s\right)V_{\pi_{\theta }}\left(s\right)\\ \; & =\sum_{s\in S}d_{\pi_{\theta }}\left(s\right)\sum_{a\in A}\pi_{\theta }Q_{\pi }\left(s,a\right), \end{array}$$

where $d_{\pi_{\theta }}\left(s\right)$ is the stationary distribution of the Markov chain for $\pi_{\theta }$. The policy gradient is then given as:

(14) $$\nabla_{\theta }J\left(\theta \right)=E_{\pi_{\theta }}\left[\nabla_{\theta }log\pi_{\theta }\left(s,a\right)Q_{\pi }\left(s,a\right)\right],$$

in which ∇ is the gradient operator.

### 3.2. MDP Formulation

To apply DRL to solve the task scheduling in edge computing, we formulate the task scheduling process as an MDP, where the state space, action space, and state transition are carefully designed. The edge server is considered as the environment, and the scheduler plays the role of the agent, which interacts with the environment and makes decisions.

#### 3.2.1. State Space

**Figure 3:**
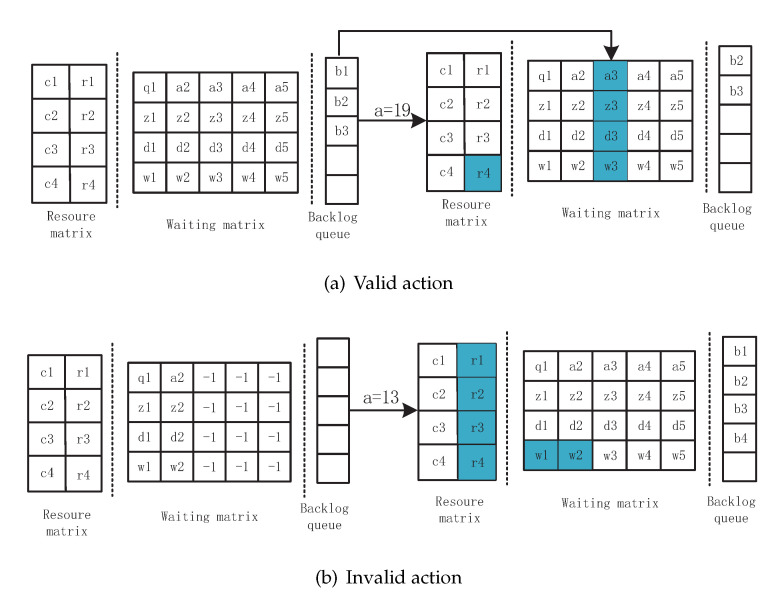
Illustration of the state transition with two examples of a valid action and an invalid action. (**a**) Valid action; the time step is frozen; (**b**) invalid action; the time step proceeds to the next time step.

The state s∈S describes the status information of the system, which is composed of three parts: the resource matrix, the task matrix, and the backlog queue length. Therefore, the state of the system can be given as:

(15) S={s|s=(V,Q,|b|)},

where V denotes the resource matrix, Q is the waiting matrix, and b indicates the backlog queue. The resource matrix represents the state of different VMs, including the processing capacity and the availability time of each VM for the next task. The waiting matrix can be observed by the scheduler, and at most one task is scheduled each scheduling time step. The tasks in the backlog queue cannot be scheduled at the current time step. As shown in Figure 3, each part of the state is elaborated as follows.

The resource matrix $V\in R^{n_{vm}\times 2}$. The first column represents the processing capacity (in MIPS) of the VMs, and the second column is the ready time for the next task that will be scheduled in the corresponding VM. For example, the VM $v_{1}$ is able to handle c1 MI per second, and r1 means that the task in the process will be completed in the future r1 time steps. The next task scheduled to individual VMs will start only if the value of r1 decreases to zero.

**Figure 4:**
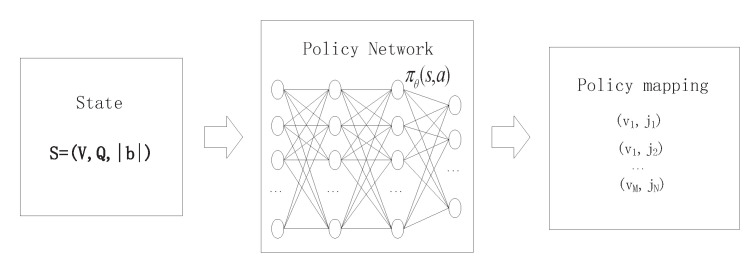
Illustration of the proposed REINFORCE network. The policy is mapped to a VM-task pair.

The tasks to be scheduled are divided into two parts: one is in the waiting slot, and the other is in the backlog queue. The tasks in the waiting slot are represented by a waiting matrix and can be scheduled at each scheduling time step. At most *O* tasks can be scheduled at each time step, and the tasks beyond *O* are stored in the backlog queue. In this case, the scheduler is able to observe the full status information of the waiting slot, while only the number of tasks at the backlog queue is visible. Therefore, the state of the waiting slot Q can be represented by a *O*-column matrix,

(16) $$Q=\;\left[q_{1},q_{2},...,q_{o}\right],$$

in which $q_{j}$ is the size of the waiting slot, which is the length of the column of the waiting matrix, as shown in Figure 4. The row indicates the task characteristics of each task, including the task type, the task size (in MI), the task expected latency, and the waiting time before being scheduled, respectively. Thus, the waiting matrix $Q\in R^{4\times O}$. In particular, when the number of accepted tasks is less than the waiting slot size $(n_{q})<O$, the empty position is padded with a fixed negative value to decrease the probability of being selected. In practice, the value can be set as $q_{j}={\left[-1,-1,-1,-1,-1\right]}^{T}$, where T denotes the transposition of the vector.

The size of the backlog size indicates the maximum number of tasks that the edge server can accept. If a task is scheduled, it leaves the slot, and the first task in the backlog queue is inserted into the slot that was just vacated. When the tasks exceed the length of the backlog queue, the extra tasks are dropped out.

#### 3.2.2. Action Space

The action space of the task scheduling includes two actions: one is to determine the execution order among *O* tasks, and the other is to assign one from *M* VMs for each task. Combining the two actions requires a large action space, resulting in the learning being too complicated. To keep the state space small, we decouple the scheduling time step *n* from the real time step *t*, and more than one task scheduling decision is made in each time step. At each time step, the time is frozen until an invalid action. The action is defined as mq+n, indicating that the task $j_{n}$ in the waiting slot is scheduled to VM $v_{m}$. Furthermore, an “invalid” action means the scheduler selects a void task, then time step *t* proceeds to the next time step t+1. Therefore, the size of the action space decreases to MO. Therefore, the scheduling process performs according to the two time steps: the scheduling time step and the real time step. At the start of each real time step, the scheduler fetches new tasks if one arrives, while the scheduling time step is the scheduler’s decision sequences. By decoupling the two time steps, the action space stays linear in MO. At each scheduling time step *n*, the action is represented as a $\left(v_{m},j_{n}\right)$ pair, which is:

(17) $$A=\left\{A_{e}\left|A_{e}=\left(v_{m},j_{n}\right)\right|\begin{array}{c} m\in \left\{-1,1,2,\ldots ,M\right\}\\ n\in \left\{-1,1,2,\ldots ,O\right\} \end{array}\right\},$$

where $\left(-1,-1\right)$ is the invalid action $A_{\psi }$, indicating a void task is scheduled, and the others are the valid action $A_{\varphi }$, indicating that task $j_{n}$ is scheduled to VM $v_{m}$.

At the beginning of the real time step *t*, new tasks are put in the waiting slot if there is an empty position; otherwise, they are put in the backlog queue. For each scheduling time step *n*, the scheduler makes a decision by observing the system state. If a valid action $A_{\varphi }$ is selected, the scheduled task is removed from the waiting slot, and the first task in the backlog queue is placed in the waiting slot that was just vacated. If an invalid action $A_{\psi }$ is selected, the time step proceeds to the next time step.

#### 3.2.3. State Transition

The state transits to the next based on the state and action (s,a). As shown in Figure 3, the cases of state transition are explained as follows.

(a) The scheduler selects a valid action, and the backlog queue is not empty. For example, in Figure 3a, a=19, that is $A_{e}=\left\{\left(v_{3},j_{3}\right)\right\}$, where the subscript indicates the index of the VM and task in the waiting slot. Then, the task $j_{3}$ is scheduled to the VM $v_{4}$ and will be executed after r4 time steps. The value of ready time r4 for the next scheduled tasks changes by pulsing the execution time to process $j_{3}$. Furthermore, the first tasks $b_{1}$ in the backlog queue are put into the position that just stores $j_{3}$ and the number of tasks of the backlog queue minus one simultaneously. It is noted that the waiting time of all the tasks stays unchanged within the same time step.
(b) An invalid action is chosen, meaning no task is scheduled and the backlog queue is empty at the current time step, as shown in Figure 3b. In this case, the time step proceeds to the next time step to accept new tasks. New tasks move to the waiting slot firstly, and the extra tasks are put into the backlog queue. The tasks are dropped if the number of new tasks is larger than the backlog queue size. Meanwhile, the waiting time in both the waiting matrix and the backlog plus one and the ready time of VM $v_{m}$ are set to a value of $max\{r_{m}-1,0\}$.
(c) The scheduler selects a valid action. After that, both the waiting slot and the backlog queue are empty. The time step goes to the next time step and fetches new tasks. In this case, only the ready time of all the VMs changes.

#### 3.2.4. Reward

As mentioned above, the objective is to maximize the LTSD, as presented in Equation (1). The reward is designed to guide the scheduler toward the goal of the optimal policy π=p(a|s). For a valid action, the reward is the ratio of the response time and the expected latency requirement. We give zero rewards if the invalid action is selected; thus, the reward function is designed as:

(18) $$r=\left\{\begin{array}{c} w_{j},a\in A_{\varphi }\\ 0,a\;\in A_{\psi } \end{array}\right..$$

### 3.3. REINFORCE Implementation

REINFORCEis a Monte Carlo policy gradient algorithm that updates the policy parameter θ based on the expected return over trajectories $\tau =\left(s^{0},a^{0},r^{1},s^{1},a^{1},r^{2},\cdots a^{T},r^{T+1},s^{T+1}\right)$. The policy gradient at each time step *t* in the trajectory of each episode is converted to:

(19) $$\nabla_{\theta }J\left(\theta \right)=E_{\pi_{\theta }}\left[\nabla_{\theta }log\pi_{\theta }\left(s^{n},a^{n}\right)G^{n}\right]$$

The parameter θ is updated according to the gradient ascent, which is:

(20) $$\theta^{n+1}=\theta^{n}+\alpha G^{n}\nabla_{\theta }log\pi_{\theta }\left(s^{n},a^{n}\right),$$

in which α is the learning rate. Equation (19) indicates that if $G^{n}$ is positive, we want to increase the log probability of selecting action $a^{n}$ in state $s^{n}$; otherwise, we decrease the log probability. The agent is thus guided to find the optimal policy.

Based on the MDP formulation in Section 3.2, at each scheduling time step *t*, the policy network accepts the system state $s^{n}=\left(V,Q,|b|\right)$ and generates the probability of selecting an action as the output. The action is selected as $\pi_{\theta }\left(a^{n}|s^{n}\right)$ and then represented as a VM-task pair $A_{e}=\left(v_{m},j_{k}\right)$. If $A_{e}=\left(-1,-1\right),$ this means that no task is scheduled at the current scheduling time step, and no reward is obtained. In this case, the real time step moves forward, and the next state is generated based on Case (b) in Section 3.2.3. In the case of a valid action (i.e., $A_{e}\in A_{\varphi }$), the real time step also proceeds to fetch new tasks if both the waiting slots q and the backlog queue are empty. The next state changes as described in Case (c) in Section 3.2.3. Additionally, if the backlog queue is not empty after a valid action is selected, the first task in the backlog queue is put into the waiting slots, and the scheduling time step adds one. The next state is obtained by Case (a) in Section 3.2.3. The states, actions, and rewards constitute an episodic trajectory to compute the cumulative reward for further training. When updating the parameter θ, the cross-entropy is applied to calculate the difference between the predicted action distribution $\pi_{\theta }\left(a|s\right)$ and the target (label) action. The outcome of the cross-entropy multiplied by the expected discounted cumulative reward is used as the loss function to optimize the policy network parameter θ. The cross-entropy is calculated as:

(21) $$L_{ce}^{n}=-y_{a}\log\left(\pi_{\theta }\left(a^{n}|s^{n}\right)\right),$$

where $y_{a}^{n}$ is the label action in scheduling time step *n*, and the final loss function of the policy network is given as:

(22) $$L_{\theta }=\frac{1}{T}\sum_{n=1}^{T}G^{n}L_{ce}^{n},$$

where $G_{n}$ is the discounted cumulative reward at time step *n* during the episode. The proposed algorithm is illustrated in Algorithm 1.
**Algorithm 1:** Task scheduling and allocation with the REINFORCE algorithm.
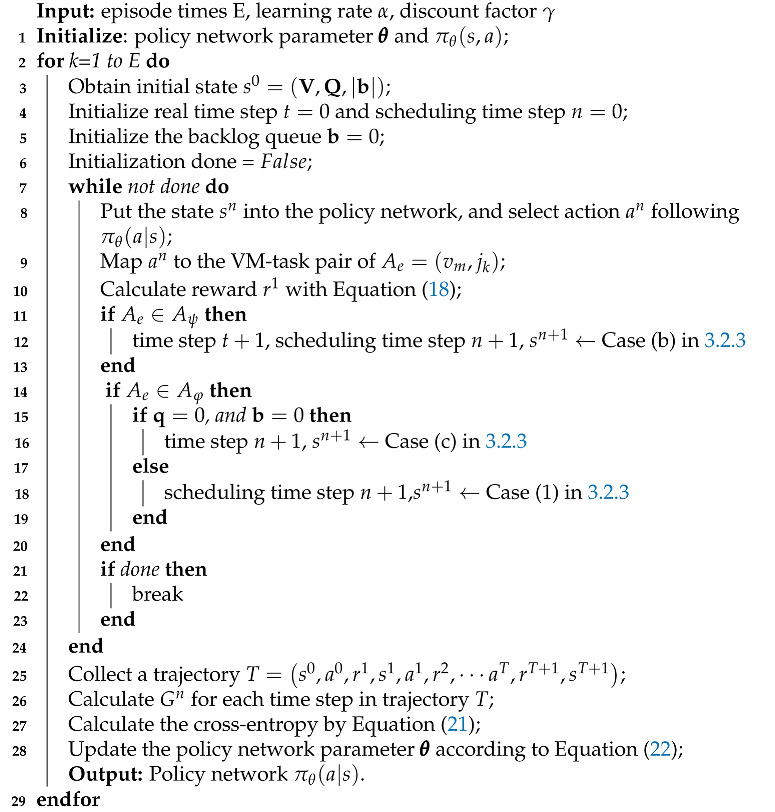


## 4. Simulation Results

In this section, numerical results are presented to evaluate the performance of the proposed task scheduling and allocation algorithm. All simulation results were obtained using Python 3 running with Pytorch. We further compared the proposed algorithm with two baselines.

### 4.1. Simulation Setting

**Figure 5:**
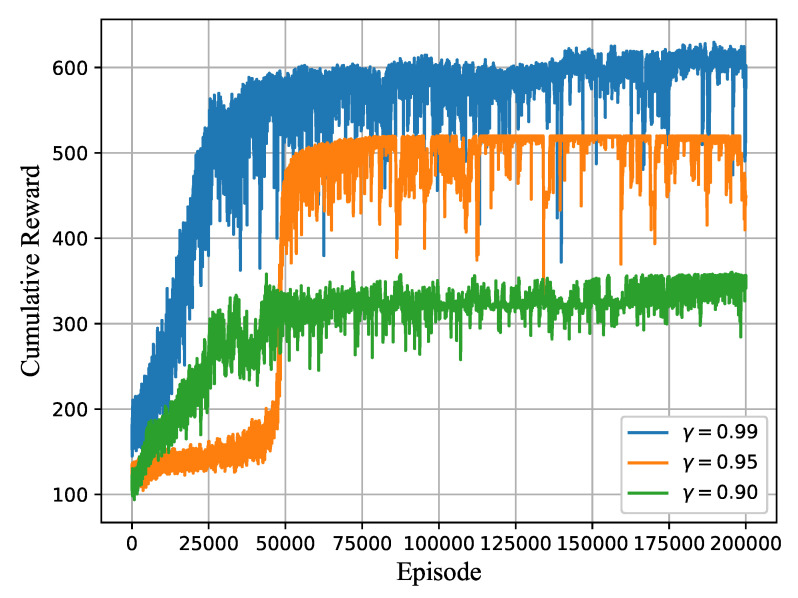
Cumulative reward per episode.

A four-layer DNN structure was applied to realize the task scheduling and allocation policy. Both hidden layers had 64 neurons, and the rectified linear unit (ReLU) was applied as the activation function. The dimension of the output layer had (M+1)×O neurons. The discount factor γ was set as 0.99 during the training, indicating that the future steps influence the current action. The learning rate was set as $10^{-4}$, and the Adam optimizer was used for gradient descent. The hyperparameters were kept fixed throughout the simulation. The detailed hyperparameter setting is shown in the table. The convergence of the proposed algorithm for different discounted factors γ during the training period is shown in Figure 5. The reward increases with the growth of γ because a higher value of γ indicates higher weights of the future rewards. In this paper, only the computational resource is considered for the task scheduling in the edge system, where the transmission delay is used to calculate the residual computational delay, so the environment parameters only include the task characteristics and the VM resources, which are described as follows.

- The tasks generated by the IoT device are sent to the BS suffer from the communication transmission and arrive at the edge server at a certain rate. We assumed that the expected latency ranges from 5 s to 10 s and the transmission delay ranges from 1 s to 5 s. The range of the task size was set as $\left[500,4000\right]$ MI. In general, any arriving rate is applicable, because it is unknown in advance and is not included in the input state feature; for convenience, the tasks arrive at the edge server according to a Poisson distribution, and the average arrival rate varies from three request/s to seven requests/s, so the task arriving interval follows an exponential distribution with $\left[0.14,0.33\right]$.
- The processing capacity of the VMs was set in the range $\left[1000,2000\right]$ MIPS.
- The size of the waiting slot was set as O=5, and the length of the backlog queue was set as |b|=5.
- There were five types of tasks in the simulation, and the task characteristics, including the size and the expected delay, are shown in Table 2.

### 4.2. Performance Evaluation

**Figure 6:**
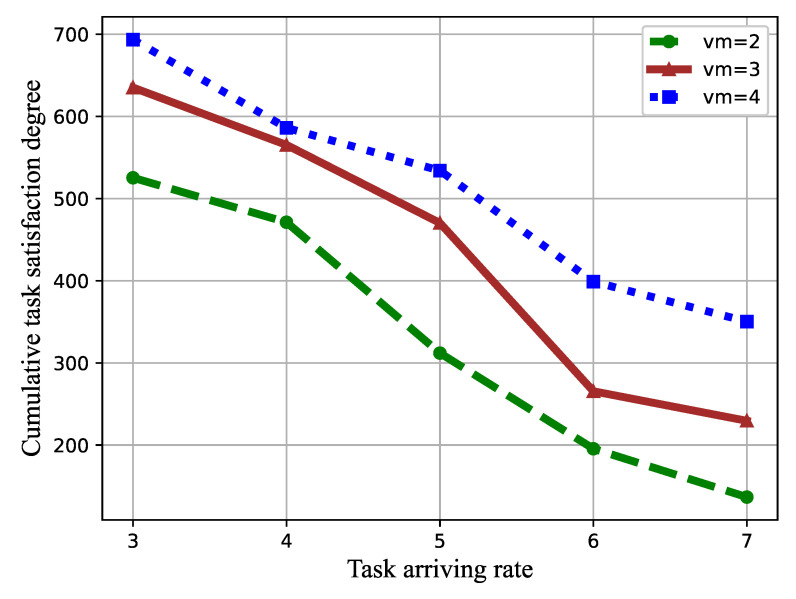
Cumulative task satisfaction degree versus task arriving rate and the number of VM.

**Figure 7:**
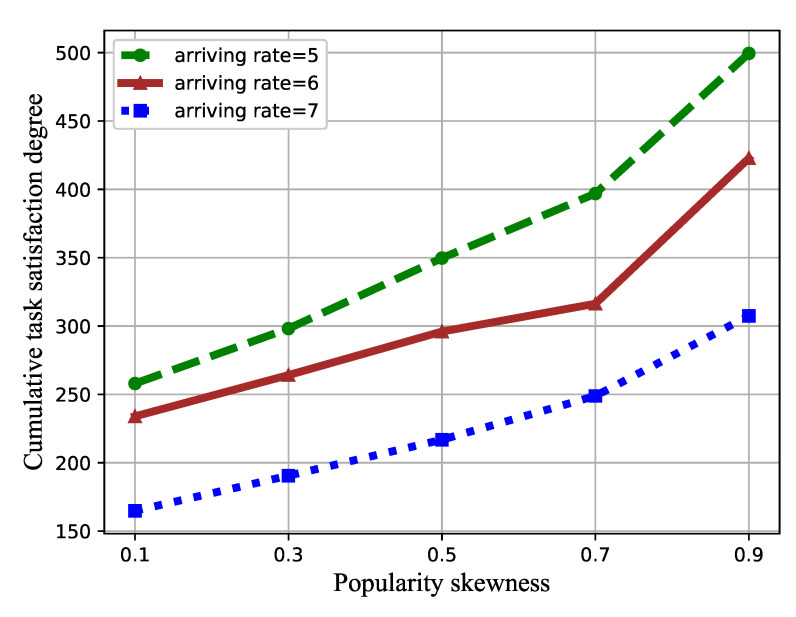
Cumulative task satisfaction degree versus the popularity skewness.

Some factors, including the task arriving rate, the number of VMs, and the task popularity skewness on the task satisfaction and the success ratio, were studied. Simulation results are shown in Figure 6 and Figure 7.

In Figure 6, we evaluate the influence of the task arriving rate λ and the number of VMs on the cumulative task satisfaction degree. The tasking arriving rate λ ranged from three to seven, and the VM number increased from three to five, while the popularity skewness was set as 0.3.

From Figure 6, it can be seen that the cumulative task satisfaction degree of the proposed DRL-based task scheduling and allocation algorithm decreases with the increment of the task arriving rate. The reason is that the higher arriving rate indicates more tasks wait to be scheduled in the edge system within the same time step, which increases the waiting time of the tasks. In terms of the number of VMs, it is apparent that the average task satisfaction degree increases when the number of VMs increases. This is because the tasks can be scheduled to more VMs, leading to a reduced waiting time.

In Figure 7, the effect of the task popularity skewness β on the task satisfaction degree is represented. The value of β increased from 0.1 to 0.9, while the number of VMs was set to three.

As shown in Figure 7, increasing β enlarges the cumulative task satisfaction degree. The popularity skewness indicates different popularities of each type of task. As β increases, the proportion of small-sized tasks increases, while the popularity of large-sized tasks decreases, which reduces the overall waiting time for tasks.

### 4.3. Performance Comparison with the Benchmark Methods

To better evaluate the performance of the proposed DRL-based task scheduling algorithm, the FCFS [20] algorithm and the SJF [21] algorithm were selected as the two benchmark methods. In both FCFS and SJF, the scheduled task is assigned to the VM with the maximum task instant reward. This means that the scheduled tasks are assigned to the VM greedily. Therefore, the two benchmarks can be expressed as greedy-FCFS and greedy-SFJ.

We compared our proposed algorithm with greedy-FCFS and greedy-SJF concerning the average task satisfaction degree and the task success ratio. The average task satisfaction degree reflects the overall quality of the algorithm, which considers the effect of a single value of each task satisfaction degree on the total task satisfaction degree. If the task’s response time is less than its expected delay requirement, that is $w_{i,j}>=1$, we say that the task is completed perfectly. The task success ratio is defined by the ratio of the number of perfectly completed tasks to the total number of tasks, which is:

(23) $$\epsilon_{s}=\frac{N_{s}}{\sum_{j\in J}N_{T}},$$

where $N_{s}$ is the number of perfectly completed tasks.

**Figure 8:**
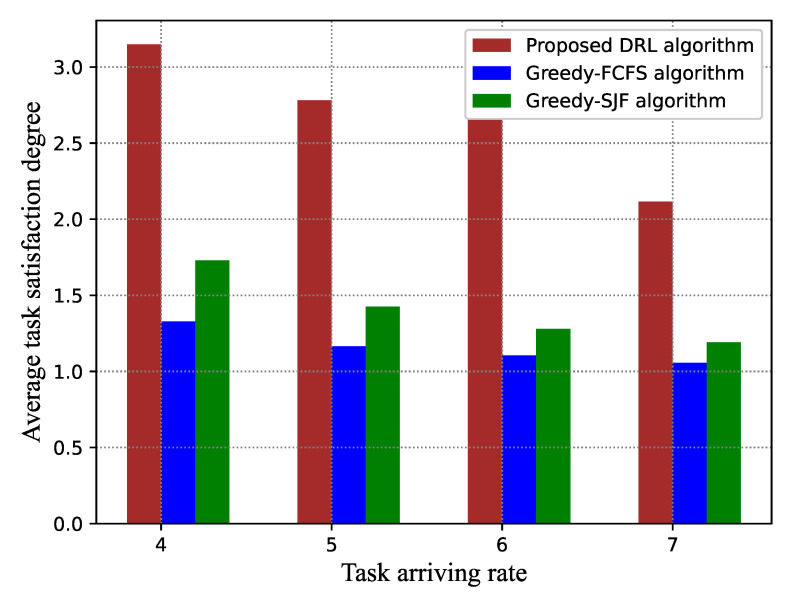
Average task satisfaction degree versus task arriving rate. FCFS, first-come-first-serve; SJF, shortest-job-first.

**Figure 9:**
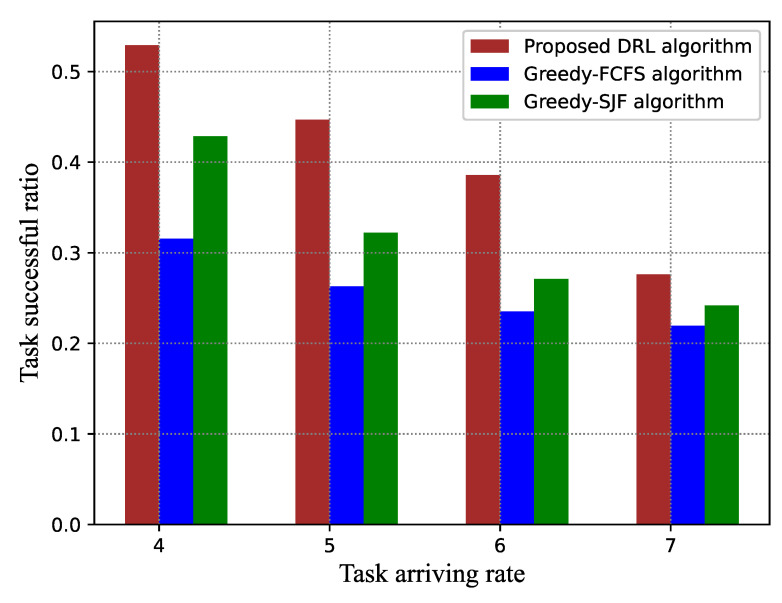
Success ratio versus task arriving rate.

Figure 8 and Figure 9 give the performance comparison under different task arriving rates λ. When the task arriving rate increases, both the average task satisfaction degree and task success ratio present a declining trend for all algorithms. Compared to the greedy-FCFS scheduling algorithm and the greedy-SJF scheduling algorithm, our proposed algorithm has a significant improvement. Specifically, the proposed algorithm can improve by around 50% and 25% the average task satisfaction degree compared to greedy-FCFS and greedy-SJF, respectively. The reason is that, in FCFS, the earlier arriving tasks are scheduled first, which will cause a long waiting time for the subsequent tasks if the earlier arriving tasks require too much CPU resource of the VMs. In greedy-SFJ, tasks with the shortest execution time have higher scheduling priority even though they arrive later, which is not good for long tasks. Neither greedy-FCFS nor greedy-SJF take into account the expected delay demand.

**Figure 10:**
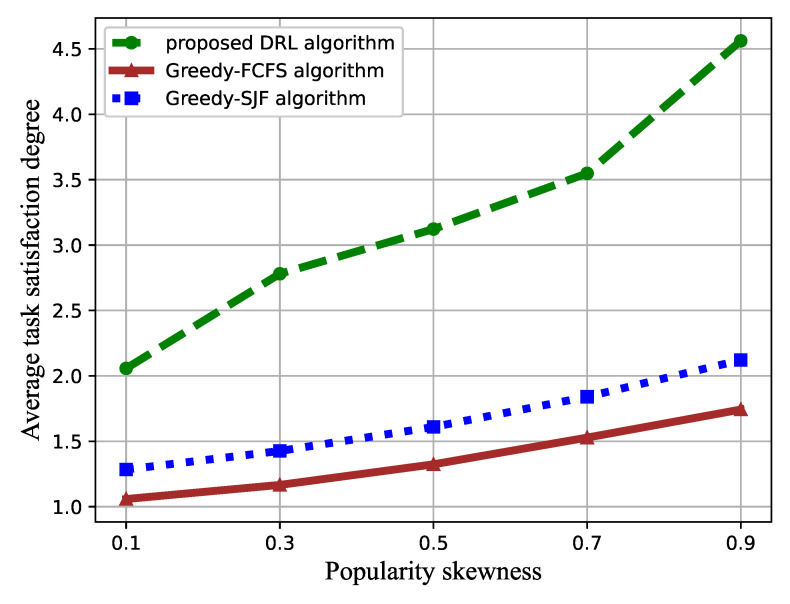
Average task satisfaction degree versus the popularity skewness.

**Figure 11:**
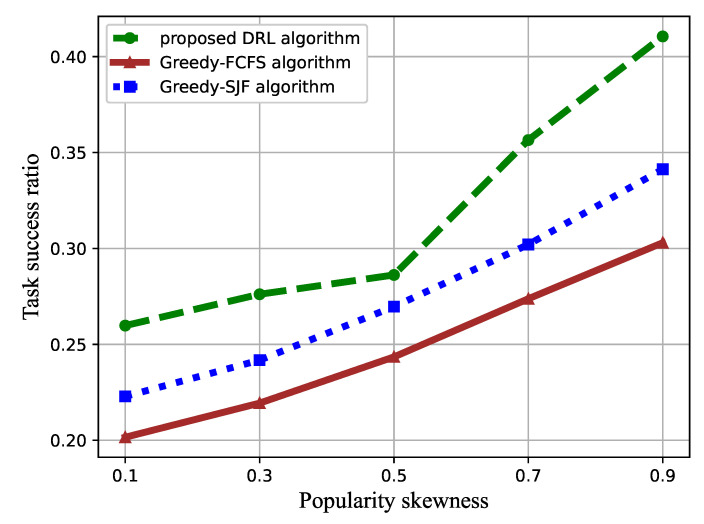
Success ratio versus the popularity skewness.

The performance comparison of the proposed DRL-based task scheduling algorithm and the baselines toward the task popularity is illustrated in Figure 10 and Figure 11. The cumulative task satisfaction degree and success ratio increase with the increment of task popularity skewness β. Additionally, from Figure 10, we can see that our proposed algorithm can significantly improve the task satisfaction. The gap enlarges with the increasing of the popularity factor value β compared to the greedy-FCFS algorithm and the greedy-SJF algorithm. This is because the small-sized tasks account for a larger proportion as β increases, resulting in a longer waiting time, leading to the performance degradation of the greedy-SJF algorithm.

The performance of the proposed algorithm, the average task satisfaction degree, and the success ratio with different VMs are illustrated in Table 3 and Table 4, where the number of VMs was set to 2, 3, and 4, respectively. The proposed algorithm achieves better performance than the greedy-FCFS and greedy-SJF methods on the task satisfaction degree and success ratio. This is because the scheduler always selects the VM that minimizes the response time for the current scheduled task without taking the future tasks into account.

## 5. Conclusions

This paper proposes the computationally-intensive task scheduling problem in the IoT edge system, where the task execution order and task allocation are jointly optimized. We formulate the optimization problems as an MDP model, where the state, action, reward, and state transition are carefully designed. To reduce the dimension of the action space, the scheduling time step is decoupled from the real time step. A policy-based deep reinforcement learning algorithm is applied to solve the MDP. It demonstrates that our proposed algorithm has good convergence performance. Moreover, extensive simulations are conducted to evaluate the cumulative task satisfaction degree and success ratio. The results show that the proposed algorithm outperforms other benchmark methods. Future work will focus on collaborative cloud computing and edge computing, where the communication delay will be taken into account.

## Figures and Tables

**Table 1:** List of notations.

Symbol	Description
$j_{i}$	the task
$a_{i}$	the arriving time of $j_{i}$
$z_{i}$	the type of $j_{i}$
$l_{i}$	the size of $j_{i}$
$d_{i}$	the expected latency of $j_{i}$
$v_{j}$	the VM
*M*	the number of VMs
*O*	the maximum tasks in the waiting slot
V	the state of the VM, with the shape of M×2
Q	the state of the waiting tasks, with the shape of 4×O
|b|	the number of tasks in the backlog queue
$tw_{i,j}$	the waiting time of $j_{i}$ scheduled to $v_{j}$
$w_{i,j}$	the task satisfaction degree of $j_{i}$ scheduled to $v_{j}$
$t_{i,j}$	the response time of $j_{i}$ scheduled to $v_{j}$

**Table 2:** Task characteristics. MI, million instructions.

Type	Size (MI)	Expect Delay (s)
1	500	5
2	1375	6
3	2250	7
4	3125	8
5	4000	10

**Table 3:** Comparison of the average task satisfaction degree.

Number of VMs	DRL	Greedy-FCFS	Greedy-SJF
VM=2	1.5175	0.9072	0.9752
VM=3	2.5502	1.4163	1.5091
VM=4	4.7276	1.9956	2.2305

**Table 4:** Comparison of the task success ratio.

Number of VMs	DRL	Greedy-FCFS	Greedy-SJF
VM=2	0.3362	0.1717	0.1740
VM=3	0.3741	0.2924	0.3204
VM=4	0.5688	0.4602	0.5613

## Data Availability

Not applicable.
